# Development of Composite Scaffolds for Load-Bearing Segmental Bone Defects

**DOI:** 10.1155/2013/458253

**Published:** 2013-07-29

**Authors:** Marcello Pilia, Teja Guda, Mark Appleford

**Affiliations:** Department of Biomedical Engineering, University of Texas at San Antonio, San Antonio, TX 78249, USA

## Abstract

The need for a suitable tissue-engineered scaffold that can be used to heal load-bearing segmental bone defects (SBDs) is both immediate and increasing. During the past 30 years, various ceramic and polymer scaffolds have been investigated for this application. More recently, while composite scaffolds built using a combination of ceramics and polymeric materials are being investigated in a greater number, very few products have progressed from laboratory benchtop studies to preclinical testing in animals. This review is based on an exhaustive literature search of various composite scaffolds designed to serve as bone regenerative therapies. We analyzed the benefits and drawbacks of different composite scaffold manufacturing techniques, the properties of commonly used ceramics and polymers, and the properties of currently investigated synthetic composite grafts. To follow, a comprehensive review of *in vivo* models used to test composite scaffolds in SBDs is detailed to serve as a guide to design appropriate translational studies and to identify the challenges that need to be overcome in scaffold design for successful translation. This includes selecting the animal type, determining the anatomical location within the animals, choosing the correct study duration, and finally, an overview of scaffold performance assessment.

## 1. Introduction

Orthopedic injuries have been a major area of concern in medicine. In the late 1990s, it was estimated that 7 million fractures occurred each year in the United States alone [1, 2] and that the total medical costs associated with all musculoskeletal conditions added up to nearly $215 billion/year [1–3]. About 800,000 bone grafting procedures conducted annually in the US contributed to these costs [4]. Currently, the bone fractures due to trauma-related injuries account for more than 1.3 million procedures in the United States alone [5, 6]. The aging of the US population as well as the increase in both frequency and severity [7] of these injuries among the elderly has resulted in a significant increase of orthopedic needs and is expected to increase in the near future [1, 4, 8–10]. In the military field, the combat wars in Afghanistan and Iraq have increased the number of trauma procedures performed on a daily basis [11]. These conflicts demonstrated the highest number of debilitating extremity wounds (54%) [11]. Indeed, out of more than 40,000 injuries and casualties sustained in these 2 wars, 82% of them resulted in at least one musculoskeletal extremity wound [12]. The extent of these incapacitating injuries includes soft tissue wounds (53%) and fractures (26%), most of which (82%) were severe open fractures [13].

## 2. Segmental Bone Defects

Natural bone has the ability to repair itself through a very well-studied healing cascade (Table 1). However, in the case of segmental bone defects (SBDs), the body cannot heal on its own, and, therefore, they represent a significant challenge in the orthopedic community. SBDs are defined as injuries in which a section of bone is completely shattered and/or absent. Usually, the size of the missing section is large enough that bone either cannot regenerate on its own (critical sized defects) or results in the formation of pseudoarthrosis (nonunions), malunions, and loss of function, making corrective surgery or additional fixation using metallic fixators a common complication [14–23].

SBDs can be brought on by trauma, disease, and age. Trauma can be related to fractures, sport, and blast injuries. Diseases include bone cancer (osteosarcoma), tumor resection and reconstruction, osteoporosis, osteoarthritis [24], generic infections, congenital deformity corrections [25], pathological degenerative bone destruction, and other degenerative diseases [20, 26, 27].  Table 2 summarizes the most common causes of SBDs and lists the risks associated with leaving these injuries untreated.

### 2.1. Bone Tissue-Engineered Scaffolds for the Treatment of SBDs

**Figure 1:**
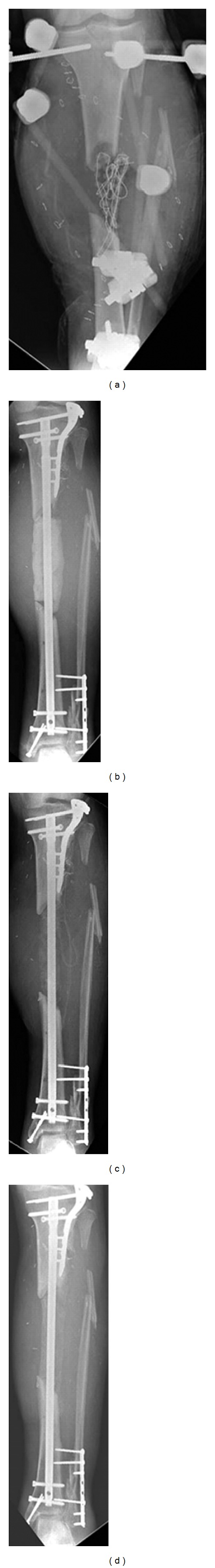
(a) Radiograph of open tibial fracture with segmental bone loss as a result of trauma injury. (b) Radiograph of the damaged tibia after intramedullary nail and internal fixation at the extremities. The defect is filled with cement spacer that had been previously impregnated with antibiotic. (c) Radiograph of the defect after 3 and (d) 4 months. Bone healing never occurred, and the fracture is considered a nonunion. Printed with permission from Dr. Steve Morgan [37].

**Figure 2:**
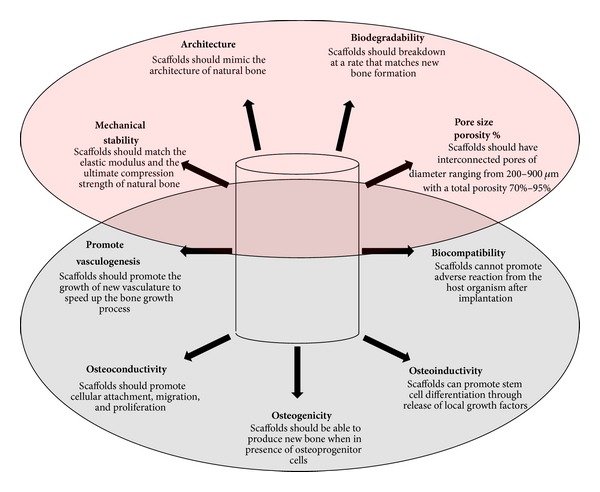
Diagram showing material (top) and biological (bottom) properties of ultimate regenerative bone scaffolds. It is necessary for engineered scaffolds to have both of these properties to promote bone growth. One class of properties alone is not sufficient to promote bone growth in a timely manner. Data originated from [21, 23, 24, 28, 38–42].

Autologous bone grafting has long been considered the gold standard for treating SBDs, but synthetic alternatives are being increasingly investigated to overcome the problems of limited availability of secondary graft sites and associated donor site morbidity [28]. Within the past two decades, the advent of tissue engineering has brought new ideas and the discovery and/or development of innovative biomaterials for bone tissue engineering purposes [8].  Figure 1 illustrates a critical size defect in which the orthopedic surgeon placed bone cement and all the necessary hardware to hold it in place. The implantation of these tissue-engineered biological constructs, also known as scaffolds, has been a major advancement in the field of orthopedics [22, 29–36]. Before understanding what the ideal bone scaffold requirements are, it is first necessary to determine the material and biological properties of these constructs. A schematic of the relationship between material and biological tissue engineering scaffolds is seen in Figure 2; Tables 3 and 4 describe the ultimate biological and material requirements that a bone tissue-engineered scaffold should possess. 

### 2.2. Bioceramics for SBD Repair

The inorganic component of bone is a carbonated Calcium Phosphate (CaP). One phase of the CaP ceramic is hydroxyapatite (HAp). Having a chemical formula (Ca_10_(PO_4_)_6_(OH)_2_), stoichiometric HAp has a structure very similar to that of natural bone [24, 26, 34, 43–45]. For this reason, synthetic CaP materials including HAp are FDA-approved and were among the most investigated materials for scaffold composition for over three decades [46–51]. CaPs differ from one another in origin, composition, morphology, and physicochemical properties. Most CaP ceramic materials are biocompatible, osteoconductive, have a bioactive surface, and can be biodegradable [18, 52], making them very well suited for bone repair, augmentation, or substitution [21, 24, 38, 39, 53]. The osteoconductive properties of CaPs support tissue ingrowth, osteoprogenitor cell growth, and the development of bone formation [54] by promoting the attachment, proliferation, differentiation, and migration of bone cells [18, 26, 39, 49]. Their surfaces also allow for a direct, adherent, and strong bond with the bone tissue that can mediate an exchange of Ca^2+^ and P ions between cell matrix and substrate [55–57]. The structural or chemical aspect of CaP ceramics can be modified. CaP coating on metals enhances osteoconductivity by stimulating rapid bone growth onto its surface [58].

### 2.3. Current Bioceramics Being Investigated

There are many different types of CaPs currently being investigated for tissue engineering purposes. HAp [23, 44, 46, 51, 59–62] is the most commonly used ceramic biomaterial in orthopedic applications because of its biocompatibility [63–65]. HAp scaffolds stimulate cell attachment, growth, and differentiation [23, 48, 66], even though they degrade at a very slow rate [67]. *β*-tricalcium phosphate (*β*-TCP) is the second most widely used CaP ceramic in bioengineering, and its alkaline nature makes it a good candidate for hybrid scaffolds to counteract the acidity resulting from polymer breakdown [8, 27, 46, 62, 68]. *β*-TCP degrades much faster than HAp and is known for its excellent biocompatibility and osteoconductivity that stimulates the proliferation and differentiation of cells [46]. Because HAp and *β*-TCP are so well suited for this application, scaffolds composed of a mixture of HAp and *β*-TCP (biphasic CaP-BCaP) are commonly reported in the literature. This combination is known for its osteoconductivity, bioactivity, biocompatibility, and degradability [38] and has drawn the attention of researchers [46]. BCaP can have a controllable degradation rate [49]. Specific ceramic properties can be found in Table 5.

The other generic classes of ceramic materials commonly used for orthopedic fixation are the bioglasses (BGs) and the CaP cements (CPC). BGs are silicon-based materials that are known for promoting bioactivity, by being able to bond to bone by developing bone-like apatite layers on their surface *in vivo* [39], promoting osteoblast differentiation, and they have similar degradation properties to HAp and *β*-TCP [25, 79]. A different material, mesoporous BG (MBG), has been reported to have greater bioactivity than BG alone [63]. Nevertheless, being a ceramic, all BGs are very brittle [39, 63]. CPCs are combinations of soft-form dicalcium phosphate (DCP) and tetracalcium phosphate (TTCP) that hardens when the two are combined [45, 80]. An advantage of self-hardening CPC is that it allows surgeons to fill in the gap between the two bone endings and conform to the shape of the defects rather than matching the gap with standard size scaffold [1, 45, 80]. Bone cements are also readily available and do not cause any major issues with either immunogenicity or disease transmission [20]. However, this material lacks a 3-dimensional porous structure and does not support bone growth well [20]. 

### 2.4. Polymer Properties

Polymeric materials are classified as natural and synthetic polymers. The former are further divided into proteins and polysaccharides. Natural polymers have weak mechanical properties but have hydrophilic surfaces that favor cell attachment and differentiation. Silk fibroin, collagen, and chitosan belong to this family. Silk is known for its biocompatibility, mechanical properties, ability to be handled in many different ways, and has relatively slow degradation rates [63]. Interconnected porosity can be achieved when the silk is prepared with organic solvent and salt leeching [66]. Collagen-I (Col-I) is the organic component of bone ECM. This makes it biocompatible and biodegradable [60], yet it lacks compressive mechanical strength and stiffness [59]. Col-I is sometimes associated with immunogenic responses from the host as well as pathogen transmission. However, the major problems associated with collagen are its cost, solubility, and lack of commercial sources [62]. Gelatin collagen is the denatured version of collagen and does not have the same drawbacks as the naturally occurring counterpart [62]. It is inexpensive, widely available, and mass producible [62]. Chitosan, a partial derivative of chitin, has received much attention because of its excellent biocompatibility and biodegradability [23]. It is only soluble at acidic pH (usually below 6.0), and when degraded it breaks down into nontoxic compounds [81].

In contrast, synthetic biodegradable polymers, such as polylactic acid (PLA), polyglycolic acid (PGA), or poly(lactic-co-glycolic acid) (PLGA), have a common disadvantage of possessing a hydrophobic surface that does not facilitate cell attachment after cell seeding [46, 63, 64]. However, these biodegradable polymers have attracted significant attention from the scientific community because of the ease with which these materials can be fabricated and because of their relative high elasticity. In general, polymers and organic scaffolds can be shaped into many different structures, creating a well suited architecture. In doing so, the mechanical properties of the material are usually lost, often resulting in very low or no compressive strength [62, 66]. These polymeric materials (PLA, PGA and PLGA) have been used as temporary extracellular matrices in bone tissue-engineering scaffolds [64] as well as sutures, thereby providing evidence of safety [27]. Nonetheless, before PLGA polymers are metabolized into their final product, they can release byproducts such as lactic acid and glycolic acid. Being acidic, these byproducts are known to cause bacteria-free inflammation or a foreign body reaction [68, 82]. There are however ways to neutralize these acids. One such way is by adding CaP ceramics, an alkaline material, to the polymeric scaffold [68]. One other disadvantage for using the PLA, PGA, or PLGA is their degradation rates. Although it has been reported that degradation of these polymers can be adjusted in the laboratory by influencing molecular mass, comonomer ratios, specimen size, configuration, and environmental conditions [8], degradation *in vivo* is difficult to control and remains among the major disadvantages for the use of these polymers [44]. 

Polycaprolactone (PCL) has been shown to have good mechanical properties and have fully interconnected pores that increase biocompatibility *in vitro *[5, 6, 38]. PCL also has a slower degradation rate than other polymers [38, 39], exists in an elastic state at room temperature, and has a low melting point of 60°, making it a good candidate for fabrication using fused deposition modeling [83]. Moreover, when implanted, PCL scaffolds do not interfere with imaging techniques [6]. However, PCL's surface is still hydrophobic and presents low affinity towards cellular attachment. This makes it necessary to find a surface modification technique to make the surface more osteoconductive [83]. Regardless, the mechanical properties of these polymeric materials alone are not similar to those of native bone [44]. PU has been shown to be polymerized in a specific manner that favors biocompatibility with human cells and tissues. It has also been shown to have adequate mechanical properties due to its hydrogen bonds within the macroparticles that hold the structure together. However, after Pierschbacher and Ruoslahti found the protein sequence needed for the cell to attach to the surface [84], cell attachment and affinity increased [82]. 

## 3. Hybrid Bone Scaffolds

Even though there are many advantages to CaPs, the drawbacks are significant and include mechanical instability, difficulty at shaping and forming them into a specific architecture, their long degradation rate, and possible bioactivity issues. The greatest concern with HAp specifically, and with ceramics in general, is that they cannot be used alone for load-bearing applications due to the brittleness (failure due to lack of plastic deformation) of these materials and the overall poor mechanical properties [20, 21, 23, 24, 26, 38, 39, 44, 59, 61, 63, 65–67, 76, 80, 85, 86]. In addition, CaP ceramics degrade very slowly [23, 26, 60, 85, 87, 88]. In contrast, fast CaP degradation is also not necessarily beneficial for tissue regeneration. It is known that *β*-TCP degrades at a much faster rate than HAp. However, faster degradation often equates to a higher level of Ca^2+^ being released, and any localized Ca^2+^ released higher than 10 mMol [89] is detrimental, including being cytotoxic at the site. It is also known that the sintering process makes CaP ceramics stronger, but compromises the surface bioactivity of the scaffold due to the overall increase in crystal size and crystallinity [38]. It has been reported that *β*-TCP is mechanically weaker than HAp and when released, the body cannot break it down into CaP, preventing strong bonding between the scaffold and the newly secreted bone [88]. In addition some researchers believe that CaPs are difficult to process [65] and are not porous enough [66]. Moreover, since it is difficult to shape sintered scaffolds as a result of their brittleness, surgeons are often forced to create an incision in the injured bone to match the scaffold when using ceramic scaffolds, thereby leading to more bone loss, trauma, and increased surgical time [1]. CPCs also cannot be used in load-bearing applications because of their low mechanical strength and lack of porous architecture that make it difficult to integrate with adjacent bones. 

By developing a hybrid scaffold composed of CaP and other polymeric materials, researchers believe that the original structure of bone could be recreated by taking advantage of the CaP's osteoconductivity as well as eliminating the brittleness of the scaffold. The combination of the osteoconductivity and the strength of CaP, in conjunction with the good workability and elasticity of biopolymers, makes hybrid composite scaffolds very good candidates for bone tissue engineering [27, 61, 66]. Hybrid scaffolds are currently being studied and developed to try to simulate the organic (Col-I) and the inorganic (HAp) portion of natural bone [44, 65]. Hybrid scaffolds consist of polymeric matrices that are paired with ceramics [79]. However, there is a wide range of polymeric materials with different properties available (Table 6). Currently, the challenge in developing a tissue-engineered scaffold with ideal properties is to find a way to combine these completely different materials together, while maintaining a porous architecture and adequate mechanical properties that favor bone formation. Even though material selection for the scaffold has a direct impact on the biological and physical properties of the construct, there are some factors contributing to the low mechanical properties that are not related to the material used. The more porous the architecture of the scaffold is, the weaker its compression strength becomes. 

### 3.1. Properties of Currently Investigated Hybrid Scaffolds

Scaffold fabrication techniques currently used include casting and particulate leaching [5, 46, 82, 90–92], gas foaming [5, 90], freeze drying [5, 25, 59, 90], electrospinning [9, 51, 90], thermally induced phase separation (TIPS) [5, 65, 90, 91], microsphere sintering [90], supercritical CO_2_ technology [93], fused deposition modeling (FDM) [5, 6], 3D printing [5], in situ precipitation [61], thermally induced phase inversion [44], selective laser sintering [40], low temperature deposition manufacturing (LDM) [68] ceramic or polymeric coating with either polymers or ceramic slurry, respectively [27], solid-liquid phase separation (SLPS) [39], and a combination of these [8, 77].

As mentioned previously, mechanical strength is considered one of the most important properties and requirements of load-bearing scaffolds. When designing a bone scaffold for tissue engineering, its mechanical properties should match that of natural bone. However, only a small percentage of investigated scaffolds in the literature are being tested for mechanical properties. Of the scaffolds-tested compression strength and/or bending and elastic modulus strength were usually investigated. However, in bone, failure due to compression is very rarely seen; most fractures are due to torsion or bending forces [94]. In contrast, the elastic modulus property determines the slope of the stress-strain curve. Ceramics have been reported to have high elastic modulus and low ductility, whereas polymers, on the contrary, have lower hardness and modulus. 

Although the concept of hybrid bone scaffold is a relatively new one, the literature has already been inundated with journal articles describing the technique and the properties of such scaffolds. A wide range of scaffolds with different properties can be found in the literature. It has been demonstrated that hybrid designs increase the overall mechanical properties of the existing scaffolds. There was a wide range of scaffolds fabricated and mechanically tested to determine their similarities to natural trabecular bone. Some implants exhibited very low compressive strength properties, 2–12 MPa, which correlate with the natural compressive strength of trabecular bone [23, 38, 61, 64, 77, 79, 86, 95]. These scaffolds should be considered for non-load-bearing applications because they also exhibited lower modulus values (up to 25 MPa), which are well below the natural range of trabecular bone (50–500 MPa). These scaffolds were generally very porous with ranges between 80, and 87%, which is essential for bone ingrowth. 

Scaffolds investigated for load-bearing applications generally had a compressive strength that ranged between 1 and 80 MPa [6, 21, 27, 44, 90, 91]. However, these samples with higher compression strength had porosities lower than 70%. The scaffold with the highest compression strength was investigated by Zhang et al. using an *in situ* precipitation technique. This HAp/PLLA composite scaffold reported compression values of 110–155 MPa [61], although no information was provided on the porosity/pore size. Nevertheless, the lack of scaffold characterization for porosity or pore size renders inconclusive whether this scaffold has tissue regenerative properties. Overall, scaffolds with lower compressive strength and modulus had a higher porosity which helped with bone growth, but scaffolds with a higher compressive strength needed for stabilization of SBDs had a lower porosity. 

## 4. *In Vivo* Load-Bearing Application Techniques

With many different *in vivo* animal studies used to investigate bone regeneration, deciding on the appropriate model and application can be challenging. First, researchers need to determine an appropriate animal model to recreate an SBD large enough that cannot self-heal. Then, proper anatomical location of the investigated scaffold in the animal should be decided, in order to recreate active loading on to the construct. At this point, an estimate length of study to see perceptible results should be determined as well as valid control groups and an adequate analysis technique. 

To date, only a few hybrid design scaffolds have been investigated *in vivo*. However, the literature is overwhelmed with the *in vivo* investigations of ceramic scaffolds [96]. This is because the concept of hybrid composite scaffolds is still relatively new, and to date, insufficient animal validation testing has been performed.  Table 7 shows the summary of the reviewed studies and differentiates them between animal choice, anatomical location, length of study, control, and analysis of results.

### 4.1. Animal Choice

With many different animals used for research, choosing an adequate model platform for the scaffolds is essential. The most important consideration is that the animal chosen should have anatomical, physiological, and pathophysiological analogies with humans. Previous models that have been used in the field vary in size. They include mice, rats, rabbits, dogs, sheep, and pigs. Once the similarities with humans are determined, it is important to keep in consideration the ability to physically provide care for many animals both during and after the testing. Other factors to keep in mind, yet not as critical, are the costs of acquisition and maintenance of the animals versus statistical size, tolerance to captivity, and ease of housing [97]. Mice and rats are used to test basic cytotoxic properties of the scaffolds, and also they are used to implant scaffolds subdermally to initiate vascularization within the construct or test osteogenicity away from an existing bone source. These are the smallest animals on which bone regeneration is tested. Rabbits, also a small animal species, show easier, faster, and more consistent bone healing. Unfortunately, this model is limited by the size and maturity of the bones and the weight of the animals [98]. Dogs have similar bone density to humans but have a series of disadvantages. Canines have faster solid bony fusion than humans, low nonunion rates, high variation between breeds, and negative perception from the public. Sheep are also very similar to humans in body weight and bone dimensions (especially long bones). The drawback is age-dependent remodeling of bone around 7 to 9 years of age. The pig is the largest animal model used for SBD regeneration. They have a very similar bone density, anatomy, and microstructure to humans. The main drawbacks, especially in load-bearing scaffolds, are that the animals are extremely large and heavy and have an accelerated rate of bone growth, which makes it difficult to differentiate between early and late remodeling [97]. 

Of the studies reviewed, the animal that was most often chosen was the rat [25, 46, 72, 77, 78]. Other studies also used rabbits [73, 76] and sheep [75]. Since hybrid scaffolds are still a relatively new concept in the bone regenerative field, it is understandable why researchers chose to test the construct on a small animal first to determine initial performance and change it accordingly. It is foreseen that this area of research will yield noticeable results in the near future to help fill the knowledge gap. 

### 4.2. Anatomical Choice for Load-Bearing SBDs

Once the animal model is chosen, the next step requires determining an appropriate surgical site that will accurately test the behavior of the bone scaffold *in vivo*. For load-bearing applications, it is necessary to use a limb or vertebral bone. This criterion allows focusing on the following bone choices: the femur or the tibia in the hind leg and the ulna/radius or humerus in the fore leg, in addition to the lumbar vertebrae. In most of the studies reviewed, the femur was the preferred anatomical choice [25, 46, 72, 73, 76–78]. However, in the only large animal model reviewed (the sheep), the tibia was chosen with a triangular defect on the medial tibia plateau [75]. The femur is the largest long bone in most animals. Since humans stand up right, the femur directly supports the body's entire load, but in animals that use four limbs for stabilization, both the humerus and the femur distribute the animal's weight. Because of these characteristics, the femur is preferred for testing load-bearing SBD model. 

After choosing the animal and the bone to test, the specific location is selected. One model involves creating a midshaft defect and placing a matching size scaffold within it [72, 78]. This model requires fixation devices to be implanted around the surgical site to maintain the two endings of the scaffold from crushing the scaffold and from moving. This model tests the mechanical properties of the scaffold and the ability to regenerate large amounts of bone. Also, this surgical site most closely resembles what occurs in clinical practice, as it gives an accurate representation of how well the scaffold will perform in a segmental setting. Extremity injuries from trauma affect all of the bone, not just parts of it. Another model involves creating only a partial defect. This is the case of rabbit radius/ulna, where only the radius is removed and the scaffold is placed next to the ulna [74]. This model might avoid the requirement of utilizing fixation devices to stabilize the animal; however, in the rabbit, this model is not truly weight bearing. 

A third model involves drilling longitudinally through the cortical bone into the cancellous bone at the medial epicondyles of femur [25, 46, 76, 77]. The advantages of the medial epicondyle of the femur model is that no surgical fixation devices are required, and since the defects span both the cortical and the trabecular bone, histological evaluation *ex vivo* can determine how the bone regenerated and whether remodeling occurred. However, if the scaffold being investigated is made to be loaded unidirectionally; then, this model will likely fail *in vivo*. When implanted in the epicondyle, the scaffold is exposed mostly to circumferential forces, and, therefore, will sustain damages before it can regenerate. Overall, this model is load protected and is seen more as a non-load-bearing model. Thus, based on the scaffold properties and architecture, the femur epicondyle might not be the best anatomical choice for this application.

### 4.3. Length of Study—Modeling versus Remodeling

The reviewed *in vivo* studies were analyzed for as little as 2 weeks [77] and for as long as 48 weeks [76]. When selecting the length of study for the *in vivo *study, there are times during which different stages of healing occur depending on the animal model which was utilized for the study. While animal models do have species-dependent bone formation rates, typically, the first 4 weeks of most animal studies are used to assess the host's response to the implant (biocompatibility). The first two months are then used for assessment of healing (modeling). Anything beyond that will be testing the remodeling process, during which the newly healed bone will gain strength. Of the reviewed studies, two rat femur models were investigated for less than 4 weeks [77, 78]. This step is of critical importance for hybrid biomaterials, since they have a high risk of causing acute inflammatory reactions depending on what polymeric material was used when constructing the scaffolds. Two other studies reviewed were performed for 1 to 2 months to assess bone healing (modeling) [25, 75], and 4 other studies instead investigated long-term remodeling of bone [46, 72, 73, 76]. Perhaps if the researcher has early *in vitro* data showing biocompatibility of the scaffold, the animal study will have different time intervals that demonstrate the healing process through early response, modeling, and remodeling processes.

### 4.4. Analysis of the SBD Models

After developing, characterizing, and implanting the scaffold in an animal, the test subjects are sacrificed so that the ability to regenerate bone can be assessed. There are a number of techniques used by researchers to investigate bone growth: histology, microcomputed tomography (*μ*-CT) analysis and bone density scans. Load-bearing scaffolds should also be investigated for their ability to gain strength while implanted. This is accomplished through the use of mechanical testing. Using histology, the tissue where the bone scaffold was implanted is processed and stained on thin sections that can be observed by microscopy. 

Most researchers use histology (either decalcified or undecalcified) to analyze new bone volume, new soft tissue formation, and area of scaffold resorbed. In hybrid scaffolds, the foreign body reaction should also be analyzed. The other common characterization performed on explanted tissues is *μ*-CT. This technique isused to analyze new bone formation, regeneration patterns, and bone density. However, because *μ*-CT reconstructions are very subjective to the operator, there is always the need for histology to support the data. The last test that should be required after investigating a load-bearing scaffold is mechanical analysis of the construct postimplantation. The excised tissues should be tested for ultimate compression, tensile strength, and elastic modulus. Surprisingly, only two long-term studies performed mechanical analysis of the defect after the sacrifice of the animals. Without this test, it is difficult to determine whether the implant was successful at recreating a load-bearing structure. 

When analyzing the performance of the scaffold, it is usually compared to positive and negative controls and preferably both. In hybrid scaffolds, a control is usually the ceramic or polymer alone and often an autograft from the same donor. It has been previously shown that there is no need to compare scaffold performance to a defect-only control since it has been shown repeatedly that an empty critical-sized defect does not heal on its own [72]. 

## 5. Summary and Perspectives

The clinical issues surrounding the treatment of load-bearing SBDs need a multipronged approach for treatment. Current strategies focus on a combination of osteoconductive substrates delivering osteoinductive growth factors and osteogenic cell sources. This review focuses on the development of composite hybrid scaffolds composed of ceramic and polymeric materials that provide the mechanical stability, structure, and calcium source required to serve as a suitable osteoconductive surface in healing SBDs. This information can be used to develop and implement *in vivo *testing of the investigated implants by selecting the appropriate animal model, the most accurate anatomical positioning in the bone, determining the length of the study, and finally the analysis of the samples. 

Clearly, there is a need for a hybrid scaffold technology with the strength of the ceramics and the elasticity of polymers that will move the field closer to a functional load-bearing scaffold. When developing the scaffolds, it is important to take into consideration the requirements of scaffolds as well as the drawbacks of the materials being used, so that the researcher can then test accordingly. This includes testing for cytotoxicity and mechanical properties. Moreover, when investigating a load-bearing scaffold, the experimental mechanical properties should always be reported. The true difference between a bone scaffold and the load-bearing scaffold resides in the mechanical properties. Surprisingly, this was not the trend seen in the literature, where many load-bearing scaffolds should have been considered safe to handle, but not to load. There are many journal articles that investigate *in vitro* hybrid bone scaffolds, but very few have moved on to *in vivo* testing. Of those few, the majority have used small animal models. It is expected that as more suitable composite scaffolds perform well *in vitro*, *in vivo* characterization will significantly increase.

After years of focusing on purely ceramic or purely polymeric scaffolds, researchers have started to consider hybrids, and despite the fact they have high potential, the field is still far from having a scaffold that can be fully loaded that supports viable bone regeneration in a reasonable time after the implant. In the best case scenario, these revolutionary scaffolds would eliminate the need of fixation devices or at least they could minimize their need. They could also be used to deliver growth factors to accelerate healing from the scaffold surface and to deliver appropriate stem cells into the wound environment to tackle critical-sized defects. The overall cost of surgeries would decrease, the healing time would be decreased significantly, and there would not be a need for multiple revision surgeries once these technologies are optimized. 

## Figures and Tables

**Table 1:** Stages of the bone-healing cascade. Adapted into a table form from [69].

Early inflammatory stage	*Hematoma development*: after injury blood permeates into the wound site, and a hematoma forms within hours.
	*Granulation tissue formation*: inflammatory cells including macrophages, monocytes, lymphocytes, and polymorphonuclear cells infiltrate the wound through blood. Fibroblasts also infiltrate the bone through the mediation of prostaglandin. This mixture results in the formation of granulation tissue.
	*Ingrowth of vascular tissue*: inflammatory cells forming the granulation tissue will also stimulate vasculogenesis.
	*Migration of mesenchymal cells*: this is the third and final process that is stimulated by the inflammatory cells found in the wound.

Repair stage	*Stroma formation to support vasculature*: during the first stage of the repair process, fibroblasts deposit stroma. This is aimed at supporting vasculature ingrowth.
	*Vascular ingrowth progress*: after the stroma is formed, vascular tissue can continue to grow and distribute nutrients to all areas of the wound.
	*Mineralization of the osteoid*: the collagen matrix is deposited by osteoblasts and subsequently is mineralized.
	*Soft callus formation*: mineralization of the osteoid leads to the formation of a soft callus around the wound area. The soft callus is a very weak structure that hardens as the callus ossifies. Any movement between bones during this stage will result in macrotrauma potentially disrupting the healing cascade.
	*Woven bone formation*: as the callus ossifies, the opposite bone extremities are bridged by woven bone formation.

Remodeling stage	*Bone returns to original structure*: the last stage of bone healing is also the longest, as it takes a minimum of 3 months and continues for the life of the bone. A factor that affects bone-remodeling time is adequate mechanical loading. This allows for the organic matrix to mineralize where needed and for bone to be resorbed where it is not.

**Table 2:** Different causes of SBDs and consequences of untreated SBDs.

Causes of SBDs	
Trauma injuries	*Blast injuries*: these types of injuries are very common amongst military personnel. The injury that results from bomb or explosive detonation is of significant nature because it not only affects the skeletal structure, but also the musculature, blood supply, and nervous system.
	*Fractures and sport injuries*: these types of fractures are often segmental and are characterized by shattered, fragmented pieces of bone. After the broken pieces are surgically removed, a gap is left between the opposite extremities.

Diseases	These include bone cancer (requiring tumor resection and reconstruction), osteoporosis, osteoarthritis, generic infections, congenital deformity corrections, and pathological degenerative bone destruction. The commonality among these diseases is that the bone is either abnormally weak or needs to be removed to prevent spreading of the disease. As a result, large segments of bone are missing or are surgically removed and need to be replaced.

Complications from untreated SBDs	
Malunion	The two fractured bone ends are able to bridge, although they are not symmetrically aligned. As a result, the new bone is still susceptible to fracture. This is commonly seen in undiagnosed/untreated fractures and leads to loss of bone function.

Nonunion (pseudarthrosis)	The two fractured bone ends are not able to heal, and no bridging occurs between them. This is seen in critical defects. Another case of nonunion is observed when there is too much movement between the bone ends (insufficient surgical fixation) and the callus is never able to ossify and harden. In many cases, surgical intervention is needed to resolve the problem and avoid further loss of bone function.

**Table 3:** Bone tissue-engineered scaffold requirements.

Biological properties	*Osteogenicity*: ability of a bone scaffold to allow bone cells to induce differentiation from uncommitted mesenchymal cells to preosteoblast lineage and to secrete and mineralize extracellular matrix.
	*Osteoconductivity*: quality of a bone scaffold having a surface that is bioactive and promotes cell attachment and migration, as well as penetration within the construct.
	*Osteoinductivity*: ability of a bone scaffold to not only support but also to initiate bone growth through growth factor or hormone release.
	*Biocompatibility*: ability of a bone scaffold to not cause an immune reaction or rejection when interacting with the body.
	*Promotes vasculogenesis*: ability of a bone scaffold to promote and/or easily allow for vasculogenesis to occur within the construct.

Material properties	*Mechanical Stability*: quality of a bone scaffold to have an ultimate compression strength that is similar to bone, while maintaining the appropriate architecture.
	*Biodegradability*: the quality of a bone scaffold to degrade naturally without creating toxic byproducts while being resorbed. The rate of degradation should match the rate of new bone formation, to avoid possible gaps in regeneration.
	*Architecture*: the quality of a bone scaffold to have a very open porous structure that is interconnected throughout the construct. This allows for greater attachment surface area, higher cell density, and easier nutrient/growth factor flow within the construct.
	*Pore size/porosity*: quality of a bone scaffold to have pore size and porosity percent similar to established guidelines. Ideal pore size ranges from 300 to 900 um in diameter, whereas overall porosity ranges from 60 to 99%.

**Table 4:** Importance of mechanical stability in segmental bone defects.

Mechanical properties	Ultimate strength of cortical bone ranges between 100 and 230 MPa. Any scaffold that does not match this strength requires surgical fixation to prevent crushing/failure of the implant. Because bone is a mechanosensor organ, it is believed that a scaffold that is loaded cyclically will benefit from faster healing time.

Surgical fixation	Current bone tissue-engineered scaffolds require surgical fixation of the fractured extremities to prevent movement between the bone endings. This allows for callus formation and ossification to occur. Surgical fixation devices include screws, hardware, and intramedullary nails. They are often made of metals, specifically titanium or titanium alloys.

Stress shielding	Condition caused by the use of surgical fixation devices in load-bearing bones. Because metals have a higher modulus and compression strength, they support nearly all of the weight. In return, the fractured bone does not sense a significant change in mechanical activity, leading to a loss in bone density over time.

**Table 5:** Significant physical properties of several of the most common bioceramics used as biomaterials [70].

Material	Density (g/cm^3^)	Tensile strength (MPa)	Compressive strength (MPa)	Modulus (GPa)	Fracture toughness *K*_1*c*_ (MPa m^1/2^)	Hardness (Knoop)	Mass fraction *α* (ppm/°C)	Fracture surface energy (J/m^2^)	Poisson's ratio	Thermal conductivity *k* (Wm^−1^K^−1^)
Hydroxyapatite	3.1	40–300	300–900	80–120	0.6–1.0	400–4500	11	2.3–20	0.28	N/A
Tricalcium phosphate	3.14	40–120	450–650	90–120	1.2	N/A	14-15	6.3–8.1	N/A	N/A
Bioglasses	1.8–2.9	20–350	800–1200	40–140	~2	4000–5000	0–14	14–50	0.21–0.24	1.5–3.6
Wollastonite glass ceramic	3.07	215	1080	118	2	N/A	N/A	N/A	N/A	N/A
SiO_2_ glass	2.2	70–120	N/A	~70	0.7–0.8	7000–7500	0.6	3.5–4.6	0.17	1.5
Al_2_O_3_	3.85–3.99	270–500	3000–5000	380–410	3–6	15000–20000	6–9	7.6–30	0.27	30
Zirconia ceramics	5.6–5.89	500–650	1850	195–210	5–8	~17000	9.8	160–350	0.27	4.11
Si_3_N_4_	3.18	600–850	500–2500	300–320	3.5–8.0	~22000	3.2	20–100	0.27	10–25
Silicon carbide	3.10–3.21	250–600	~650	350–450	3–6	~27000	4.3–5.5	22–40	0.24	100–150
Graphite	1.5–2.25	5.6–25	35–80	3.5–12	1.9–3.5	N/A	1–3	~500	0.3	120–180
Multiceramics	1.5–2.2	200–700	330–360	25–40	N/A	N/A	1–10	N/A	0.3	2.5–420
Carbon fiber	1.5–1.8	400–5000	330–360	200–700	N/A	N/A	N/A	N/A	N/A	N/A
Glassy carbon	1.4–1.6	150–250	~690	25–40	N/A	8200	2.2–3.2	N/A	N/A	N/A

**Table 6:** Significant physical properties of several of the most common polymers [71].

Polymer	Glass melting point Tm	Glass transition point	Biodegradation time (months)	Compressive strength	Tensile strength	Modulus
PE				0.1–1.0	0.4–4.0	170
PMMA				3.5	1.5	160
PDLLA	Amorphous	55–60	12–16	Pellet: 150*	Film or disk: 29–35	Film or disk: 1.9–2.4
PLLA	173–178	60–65	>24	Pellet: 120*	Film or disk: 28–50 Fiber: 870–2300	Film or disk: 1.2–3.0 Fiber: 10–16
PGA	225–230	35–40	6–12		Fiber: 340–920	Fiber: 7–14
PLGA	Amorphous	45–55	Adjustable: 1–12		41.4–55.2	1.4–2.8
PPF		N/A	Bulk	30*	2	
PCL	58	−72	>24			
PHA and blends	120–177	−2 to 4	Bulk		20–43	
Poly(anhydrides)	150–200		Surface	40*	25–27	0.14–1.4
Poly(ortho esters)	30–100		Surface	16*		2.5–4.4
Polyphosphazene	−66 to 50	242	Surface			

**Table:** (a)

Author, year	Ceramic material	Polymeric material	Animal choice	Anatomical choice	Length of study	Time points	Sample size	Defect size	Scaffold size	Control
Cao and Kuboyama, 2010 [46]	*β*-TCP	PGA	Sprague-Dawley rats	Femur; Medial epicondyle	12 weeks	0, 14, 30, and 90 days	5/time points/group	3 mm diameter; 2 mm depth	N/A	(+) HAp (−) no implant

Chu et al., 2007 [72]	TCP	PPF	Long Evans rats	Femur	15 weeks	6 and 15 weeks	4 or 7/time point/group	5 mm	OD: 4 mm—ID: 2 mm; Height 5 mm	No BMP

Jegoux et al., 2008 [73]	BCaP	Collagen	New Zealand white rabbit and beagle dogs	Femur	18 weeks	18 weeks	6 rabbits, 6 dogs	20 mm	5 × 5 × 5 mm	

Guda et al., 2011 [74]	HAp		New Zealand white rabbit	Radial diaphysis	8 weeks	4 and 8 weeks	12/time point/group	10 mm		(+) autograft (−) no implant

Ignatius et al., 2001 [75]	*β*-TCP	PLA	Merino sheep	Tibia	8 weeks	6, 12, and 24 months	6/time point/group	N/A	24 mm length, 14 mm wide, 6 mm thick	(+) TCP (−) autograft

Jayabalan et al., 2010 [76]	HAp	HT-PPFhm	Rabbit	Femur	48 weeks	12, 24, and 48 weeks	2/time point	4 mm diameter; 2 mm depth	N/A	(−) no implant

Lickorish et al., 2007 [77]	TTCP and DCPA	PLGA	Wistar rats	Femur	2 weeks	2 weeks	N/A	2.3 mm diameter	2 mm diameter	PLGA scaffold

Rai et al., 2010 [78]	TCP	PCL	CBH/Rnu rats	Femur	3 weeks	3 weeks	6/time point	8 mm	8 mm high, 4 mm diameter	(−) non-seeded

Xu et al., 2011 [25]	Bioglass	Collagen-phosphatidylserine	Sprague-Dawley rats	Femur	6 weeks	3 day, 3 and 6 weeks	3/time point/group	3.5 mm diameter; 4.5 mm diameter	N/A	No phosphatidylserine

**Table:** (b)

Author, year	Type of testing	Type of histology	Histological parameters analyzed	*μ*-CT parameters analyzed	Mechanical testing
Cao and Kuboyama, 2010 [46]	*μ*-CT, bone mineral density (new bone quantity), histology, and biodegradation	Decalcified histology	Area of material in defect, new bone volume/total volume percent material biodegradation	Bone reformation	No

Chu et al., 2007 [72]	Radiograph, *μ*-CT, and histology	MMC histology	New bone formation	Callus and scaffold volumetric bone mineral density	Four-point bending

Jegoux et al., 2008 [73]	Polarized Light; *μ*-CT, SEM	Glycol methacrylate	Used thick histology sections for observation under poler	Bioceramic, newly formed bone at the center, and superior and inferior quarter of the implant	No

Guda et al., 2011 [74]	Radiograph, *μ*-CT, histology	MMC histology	Mineralized bone, fibrous tissue	Bone regeneration patterns, bone density, bone growth profiles, and overall bone volume	Four-point bending

Ignatius et al., 2001 [75]	Mechanical, histology	Undecalcified histology	New bone formation, new soft tissue formation, remaining implant components	No	Compression of 5 × 5 × 3 mm cubes

Jayabalan et al., 2010 [76]	Histology	Resin histology	Foreign body giant cell, bone growth	No	No

Lickorish et al., 2007 [77]	Histology	Decalcified histology	Fibrous tissue formation, bone ingrowth, foreign body reaction	No	No

Rai et al., 2010 [78]	Radiograph, *μ*-CT, histology	Decalcified histology	Presence of fibroblasts, chondrocytes, woven bone	New bone formation	No

Xu et al., 2011 [25]	Histology; radiography	Decalcified histology	Inflammatory reaction, new bone formation, scaffold resorption	No	No

## References

[1] Xu HHK, Weir MD, Simon CG (2008). Injectable and strong nano-apatite scaffolds for cell/growth factor delivery and bone regeneration. *Dental Materials*.

[2] Praemer A, Furner S, Rice DP (1999). *Musculoskeletal Conditions in the United Statesed*.

[3] Ambrosio AM, Sahota JS, Khan Y, Laurencin CT (2001). A novel amorphous calcium phosphate polymer ceramic for bone repair: I. Synthesis and characterization. *Journal of Biomedical Materials Research*.

[4] Laurencin CT, Ambrosio AMA, Borden MD, Cooper JA (1999). Tissue engineering: orthopedic applications. *Annual Review of Biomedical Engineering*.

[5] Chim H, Hutmacher DW, Chou AM (2006). A comparative analysis of scaffold material modifications for load-bearing applications in bone tissue engineering. *International Journal of Oral and Maxillofacial Surgery*.

[6] Gibson I, Savalani MM, Lam CX (2009). Towards a medium/high load-bearing scaffold fabrication system. *Tsinghua Science and Technology*.

[7] An YH (2002). *Internal Fixation in Osteoporotic Bone*.

[8] Yang Y, Zhao Y, Tang G, Li H, Yuan X, Fan Y (2008). In vitro degradation of porous poly(l-lactide-co-glycolide)/*β*-tricalcium phosphate (PLGA/*β*-TCP) scaffolds under dynamic and static conditions. *Polymer Degradation and Stability*.

[9] Yang F, Wolke JGC, Jansen JA (2008). Biomimetic calcium phosphate coating on electrospun poly(*ε*-caprolactone) scaffolds for bone tissue engineering. *Chemical Engineering Journal*.

[10] Hench LL, Polak JM (2002). Third-generation biomedical materials. *Science*.

[11] Owens BD, Kragh JF, Wenke JC, Macaitis J, Wade CE, Holcomb JB (2008). Combat wounds in operation iraqi freedom and operation enduring freedom. *Journal of Trauma*.

[12] Devore DI, Walters TJ, Christy RJ (2011). For combat wounded: extremity trauma therapies from the USAISR. *Military Medicine*.

[13] Owens BD, Kragh JF, Macaitis J, Svoboda SJ, Wenke JC (2007). Characterization of extremity wounds in operation Iraqi freedom and operation enduring freedom. *Journal of Orthopaedic Trauma*.

[14] Brandoff JF, Silber JS, Vaccaro AR (2008). Contemporary alternatives to synthetic bone grafts for spine surgery. *American Journal of Orthopedics*.

[15] Buma P, Schreurs W, Verdonschot N (2004). Skeletal tissue engineering—from in vitro studies to large animal models. *Biomaterials*.

[16] Cowan CM, Soo C, Ting K, Wu B (2005). Evolving concepts in bone tissue engineering. *Current Topics in Developmental Biology*.

[17] Drosse I, Volkmer E, Capanna R, Biase PD, Mutschler W, Schieker M (2008). Tissue engineering for bone defect healing: an update on a multi-component approach. *Injury*.

[18] LeGeros RZ (2002). Properties of osteoconductive biomaterials: calcium phosphates. *Clinical Orthopaedics and Related Research*.

[19] Mistry AS, Mikos AG (2005). Tissue engineering strategies for bone regeneration. *Advances in Biochemical Engineering/Biotechnology*.

[20] Weinand C, Pomerantseva I, Neville CM (2006). Hydrogel-*β*-TCP scaffolds and stem cells for tissue engineering bone. *Bone*.

[21] Puértolas JA, Vadillo JL, Sánchez-Salcedo S, Nieto A, Gómez-Barrena E, Vallet-Regí M (2011). Compression behaviour of biphasic calcium phosphate and biphasic calcium phosphate-agarose scaffolds for bone regeneration. *Acta Biomaterialia*.

[22] Lu Z, Roohani-Esfahani S-I, Wang G, Zreiqat H (2012). Bone biomimetic microenvironment induces osteogenic differentiation of adipose tissue-derived mesenchymal stem cells. *Nanomedicine: Nanotechnology, Biology, and Medicine*.

[23] Fan J, Bi L, Wu T A combined chitosan/nano-size hydroxyapatite system for the controlled release of icariin. *Journal of Materials Science. Materials in Medicine*.

[24] Baino F, Verné E, Vitale-Brovarone C (2009). 3-D high-strength glass-ceramic scaffolds containing fluoroapatite for load-bearing bone portions replacement. *Materials Science and Engineering C*.

[25] Xu C, Su P, Chen X (2011). Biocompatibility and osteogenesis of biomimetic Bioglass-Collagen-Phosphatidylserine composite scaffolds for bone tissue engineering. *Biomaterials*.

[26] Kim S-S, Sun Park M, Jeon O, Yong Choi C, Kim B-S (2006). Poly(lactide-co-glycolide)/hydroxyapatite composite scaffolds for bone tissue engineering. *Biomaterials*.

[27] Kang Y, Scully A, Young DA (2011). Enhanced mechanical performance and biological evaluation of a PLGA coated *β*-TCP composite scaffold for load-bearing applications. *European Polymer Journal*.

[28] Khadka A, Li J, Li Y, Gao Y, Zuo Y, Ma Y (2011). Evaluation of hybrid porous biomimetic nano-hydroxyapatite/polyamide 6 and bone marrow-derived stem cell construct in repair of calvarial critical size defect. *Journal of Craniofacial Surgery*.

[29] Bouler JM, Trecant M, Delecrin J, Royer J, Passuti N, Daculsi G (1996). Macroporous biphasic calcium phosphate ceramics: influence of five synthesis parameters on compressive strength. *Journal of Biomedical Materials Research*.

[30] Deville S, Saiz E, Nalla RK, Tomsia AP (2006). Freezing as a path to build complex composites. *Science*.

[31] Deville S, Saiz E, Tomsia AP (2006). Freeze casting of hydroxyapatite scaffolds for bone tissue engineering. *Biomaterials*.

[32] Gbureck U, Hölzel T, Doillon CJ, Müller FA, Barralet JE (2007). Direct printing of bioceramic implants with spatially localized angiogenic factors. *Advanced Materials*.

[33] Li SH, De Wijn JR, Layrolle P, De Groot K (2002). Synthesis of macroporous hydroxyapatite scaffolds for bone tissue engineering. *Journal of Biomedical Materials Research*.

[34] Montufar EB, Traykova T, Gil C (2010). Foamed surfactant solution as a template for self-setting injectable hydroxyapatite scaffolds for bone regeneration. *Acta Biomaterialia*.

[35] Studart AR, Gonzenbach UT, Tervoort E, Gauckler LJ (2006). Processing routes to macroporous ceramics: a review. *Journal of the American Ceramic Society*.

[36] Vitale-Brovarone C, Miola M, Balagna C, Verné E (2008). 3D-glass-ceramic scaffolds with antibacterial properties for bone grafting. *Chemical Engineering Journal*.

[37] Hak DJ (2011). Management of aseptic tibial nonunion. *Journal of the American Academy of Orthopaedic Surgeons*.

[38] Roohani-Esfahani S-I, Nouri-Khorasani S, Lu Z, Appleyard R, Zreiqat H (2010). The influence hydroxyapatite nanoparticle shape and size on the properties of biphasic calcium phosphate scaffolds coated with hydroxyapatite-PCL composites. *Biomaterials*.

[39] Fabbri P, Cannillo V, Sola A, Dorigato A, Chiellini F (2010). Highly porous polycaprolactone-45S5 Bioglass scaffolds for bone tissue engineering. *Composites Science and Technology*.

[40] Tan KH, Chua CK, Leong KF (2003). Scaffold development using selective laser sintering of polyetheretherketone-hydroxyapatite biocomposite blends. *Biomaterials*.

[41] Helm GA, Dayoub H, Jane JA (2001). Gene-based therapies for the induction of spinal fusion. *Neurosurgical Focus*.

[42] Helm GA, Dayoub H, Jane JA (2001). Bone graft substitutes for the promotion of spinal arthrodesis. *Neurosurgical Focus*.

[43] Tanase CE, Popa MI, Verestiuc L (2011). Biomimetic bone scaffolds based on chitosan and calcium phosphates. *Materials Letters*.

[44] Wang H, Li Y, Zuo Y, Li J, Ma S, Cheng L (2007). Biocompatibility and osteogenesis of biomimetic nano-hydroxyapatite/polyamide composite scaffolds for bone tissue engineering. *Biomaterials*.

[45] Xu HHK, Weir MD, Burguera EF, Fraser AM (2006). Injectable and macroporous calcium phosphate cement scaffold. *Biomaterials*.

[46] Cao H, Kuboyama N (2010). A biodegradable porous composite scaffold of PGA/*β*-TCP for bone tissue engineering. *Bone*.

[47] Teo WE, Liao S, Chan C, Ramakrishna S (2011). Fabrication and characterization of hierarchically organized nanoparticle-reinforced nanofibrous composite scaffolds. *Acta Biomaterialia*.

[48] Zhao K, Tang Y-F, Qin Y-S, Wei J-Q (2011). Porous hydroxyapatite ceramics by ice templating: freezing characteristics and mechanical properties. *Ceramics International*.

[49] Ramay HRR, Zhang M (2004). Biphasic calcium phosphate nanocomposite porous scaffolds for load-bearing bone tissue engineering. *Biomaterials*.

[50] Bernardo E, Colombo P, Cacciotti I (2012). Porous wollastonite-hydroxyapatite bioceramics from a preceramic polymer and micro- or nano-sized fillers. *Journal of the European Ceramic Society*.

[51] Luong ND, Moon I-S, Lee DS, Lee Y-K, Nam J-D (2008). Surface modification of poly(l-lactide) electrospun fibers with nanocrystal hydroxyapatite for engineered scaffold applications. *Materials Science and Engineering C*.

[52] Lerouxel E, Weiss P, Giumelli B (2006). Injectable calcium phosphate scaffold and bone marrow graft for bone reconstruction in irradiated areas: an experimental study in rats. *Biomaterials*.

[53] Xiong L, Xiong D, Yang Y, Jin J (2011). Friction, wear, and tensile properties of vacuum hot pressing crosslinked UHMWPE/nano-HAP composites. *Journal of Biomedical Materials Research B*.

[54] Urist MR (1965). Bone: formation by autoinduction. *Science*.

[55] Hench LL (1991). Bioceramics: from concept to clinic. *Journal of the American Ceramic Society*.

[56] Hench LL, Splinter RJ, Allen WC (1972). Bonding mechanisms at the interface of ceramic prosthetic materials. *Journal of Biomedical Materials Research*.

[57] Osborn JF, Newesely H (1980). The material science of calcium phosphate ceramics. *Biomaterials*.

[58] Dey A, Nandi SK, Kundu B (2011). Evaluation of hydroxyapatite and *β*-tri calcium phosphate microplasma spray coated pin intra-medullary for bone repair in a rabbit model. *Ceramics International*.

[59] Kane RJ, Roeder RK (2012). Effects of hydroxyapatite reinforcement on the architecture and mechanical properties of freeze-dried collagen scaffolds. *Journal of the Mechanical Behavior of Biomedical Materials*.

[60] Li J, Chen Y, Yin Y, Yao F, Yao K (2007). Modulation of nano-hydroxyapatite size via formation on chitosan-gelatin network film in situ. *Biomaterials*.

[61] Zhang CY, Lu H, Zhuang Z, Wang XP, Fang QF (2010). Nano-hydroxyapatite/poly(L-lactic acid) composite synthesized by a modified in situ precipitation: preparation and properties. *Journal of Materials Science: Materials in Medicine*.

[62] Bakhtiari L, Rezaie HR, Hosseinalipour SM, Shokrgozar MA (2010). Investigation of biphasic calcium phosphate/gelatin nanocomposite scaffolds as a bone tissue engineering. *Ceramics International*.

[63] Wu C, Zhang Y, Zhu Y, Friis T, Xiao Y (2010). Structure-property relationships of silk-modified mesoporous bioglass scaffolds. *Biomaterials*.

[64] Asefnejad A, Behnamghader A, Khorasani TM, Farsadzadeh B (2011). Polyurethane/fluor-hydroxyapatite nanocomposite scaffolds for bone tissue engineering. part I: morphological, physical, and mechanical characterization. *International Journal of Nanomedicine*.

[65] Wei G, Ma PX (2004). Structure and properties of nano-hydroxyapatite/polymer composite scaffolds for bone tissue engineering. *Biomaterials*.

[66] Bhumiratana S, Grayson WL, Castaneda A (2011). Nucleation and growth of mineralized bone matrix on silk-hydroxyapatite composite scaffolds. *Biomaterials*.

[67] Polini A, Pisignano D, Parodi M, Quarto R, Scaglione S (2011). Osteoinduction of human mesenchymal stem cells by bioactive composite scaffolds without supplemental osteogenic growth factors. *PLoS One*.

[68] Yang F, Cui W, Xiong Z, Liu L, Bei J, Wang S (2006). Poly(l,l-lactide-co-glycolide)/tricalcium phosphate composite scaffold and its various changes during degradation in vitro. *Polymer Degradation and Stability*.

[69] Kalfas IH (2001). Principles of bone healing. *Neurosurgical Focus*.

[70] Carter CB, Norton MG (2007). *Ceramic Materials: Science and Engineering*.

[71] Rezwan K, Chen QZ, Blaker JJ, Boccaccini AR (2006). Biodegradable and bioactive porous polymer/inorganic composite scaffolds for bone tissue engineering. *Biomaterials*.

[72] Chu T-MG, Warden SJ, Turner CH, Stewart RL (2007). Segmental bone regeneration using a load-bearing biodegradable carrier of bone morphogenetic protein-2. *Biomaterials*.

[73] Jegoux F, Aguado E, Cognet R (2008). Repairing segmental defect with a composite associating collagen membrane and MBCP combined with total bone marrow graft in irradiated bone defect: an experimental study in rabbit. *Key Engineering Materials*.

[74] Guda T, Walker JA, Pollot BE (2011). *In vivo* performance of bilayer hydroxyapatite scaffolds for bone tissue regeneration in the rabbit radius. *Journal of Materials Science: Materials in Medicine*.

[75] Ignatius AA, Betz O, Augat P, Claes LE (2001). *In vivo* investigations on composites made of resorbable ceramics and poly(lactide) used as bone graft substitutes. *Journal of Biomedical Materials Research*.

[76] Jayabalan M, Shalumon KT, Mitha MK, Ganesan K, Epple M (2010). Effect of hydroxyapatite on the biodegradation and biomechanical stability of polyester nanocomposites for orthopaedic applications. *Acta Biomaterialia*.

[77] Lickorish D, Guan L, Davies JE (2007). A three-phase, fully resorbable, polyester/calcium phosphate scaffold for bone tissue engineering: evolution of scaffold design. *Biomaterials*.

[78] Rai B, Lin JL, Lim ZXH, Guldberg RE, Hutmacher DW, Cool SM (2010). Differences between in vitro viability and differentiation and *in vivo* bone-forming efficacy of human mesenchymal stem cells cultured on PCL-TCP scaffolds. *Biomaterials*.

[79] Barroca N, Daniel-Da-Silva AL, Vilarinho PM, Fernandes MHV (2010). Tailoring the morphology of high molecular weight PLLA scaffolds through bioglass addition. *Acta Biomaterialia*.

[80] Xu HHK, Simon CG (2004). Self-hardening calcium phosphate composite scaffold for bone tissue engineering. *Journal of Orthopaedic Research*.

[81] Yoshida A, Miyazaki T, Ishida E, Ashizuka M (2004). Preparation of bioactive chitosan-hydroxyapatite nanocomposites for bone repair through mechanochemical reaction. *Materials Transactions*.

[82] Huang Y, Ren J, Ren T (2010). Bone marrow stromal cells cultured on poly (lactide-co-glycolide)/nano-hydroxyapatite composites with chemical immobilization of Arg-Gly-Asp peptide and preliminary bone regeneration of mandibular defect thereof. *Journal of Biomedical Materials Research A*.

[83] Choong C, Triffitt JT, Cui ZF (2004). Polycaprolactone scaffolds for bone tissue engineering: effects of a calcium phosphate coating layer on osteogenic cells. *Food and Bioproducts Processing*.

[84] Pierschbacher MD, Ruoslahti E (1984). Cell attachment activity of fibronectin can be duplicated by small synthetic fragments of the molecule. *Nature*.

[85] Fernandez JM, Molinuevo MS, Cortizo MS, Cortizo AM (2011). Development of an osteoconductive PCL-PDIPF-hydroxyapatite composite scaffold for bone tissue engineering. *Journal of Tissue Engineering and Regenerative Medicine*.

[86] Roohani-Esfahani SI, Lu ZF, Li JJ, Ellis-Behnke R, Kaplan DL, Zreiqat H (2012). Effect of self-assembled nanofibrous silk/polycaprolactone layer on the osteoconductivity and mechanical properties of biphasic calcium phosphate scaffolds. *Acta Biomaterialia*.

[87] Wei J, Jia J, Wu F (2010). Hierarchically microporous/macroporous scaffold of magnesium-calcium phosphate for bone tissue regeneration. *Biomaterials*.

[88] Wu S-C, Hsu H-C, Hsu S-K, Wang W-H, Ho W-F (2011). Preparation and characterization of four different compositions of calcium phosphate scaffolds for bone tissue engineering. *Materials Characterization*.

[89] Torcasio A, Van Lenthe GH, Van Oosterwyck H (2008). The importance of loading frequency, rate and vibration for enhancing bone adaptation and implant osseointegration. *European Cells and Materials*.

[90] Nukavarapu SP, Kumbar SG, Brown JL (2008). Polyphosphazene/nano-hydroxyapatite composite microsphere scaffolds for bone tissue engineering. *Biomacromolecules*.

[91] Nemati Hayati A, Rezaie HR, Hosseinalipour SM (2011). Preparation of poly(3-hydroxybutyrate)/nano-hydroxyapatite composite scaffolds for bone tissue engineering. *Materials Letters*.

[92] Misra SK, Ansari TI, Valappil SP (2010). Poly(3-hydroxybutyrate) multifunctional composite scaffolds for tissue engineering applications. *Biomaterials*.

[93] Mou Z-L, Zhao L-J, Zhang Q-A, Zhang J, Zhang Z-Q (2011). Preparation of porous PLGA/HA/collagen scaffolds with supercritical CO_2_ and application in osteoblast cell culture. *Journal of Supercritical Fluids*.

[94] Pereira FAM, Morais JJL, Dourado N, De Moura MFSF, Dias MIR (2011). Fracture characterization of bone under mode II loading using the end loaded split test. *Journal of the Mechanical Behavior of Biomedical Materials*.

[95] Liu H, Zhang L, Shi P, Zou Q, Zuo Y, Li Y (2010). Hydroxyapatite/polyurethane scaffold incorporated with drug-loaded ethyl cellulose microspheres for bone regeneration. *Journal of Biomedical Materials Research B*.

[96] Guda T, Oh S, Appleford MR, Ong JL (2012). Bilayer hydroxyapatite scaffolds for maxillofacial bone tissue engineering. *The International Journal of Oral & Maxillofacial Implants*.

[97] Reichert JC, Saifzadeh S, Wullschleger ME (2009). The challenge of establishing preclinical models for segmental bone defect research. *Biomaterials*.

[98] Liebschner MAK (2004). Biomechanical considerations of animal models used in tissue engineering of bone. *Biomaterials*.

